# Inflammatory Profile of Awake Function-Controlled Craniotomy and Craniotomy under General Anesthesia

**DOI:** 10.1155/2009/670480

**Published:** 2009-06-08

**Authors:** Markus Klimek, Jaap W. Hol, Stephan Wens, Claudia Heijmans-Antonissen, Sjoerd Niehof, Arnaud J. Vincent, Jan Klein, Freek J. Zijlstra

**Affiliations:** ^1^Department of Anesthesiology, Erasmus MC, P. O. Box 2040, 3000 CA Rotterdam, The Netherlands; ^2^Department of Neurosurgery, Erasmus MC, P. O. Box 2040, 3000 CA Rotterdam, The Netherlands

## Abstract

*Background*. Surgical stress triggers an inflammatory response and releases mediators into human plasma such as interleukins (ILs). Awake craniotomy and craniotomy performed under general anesthesia may be associated with different levels of stress. Our aim was to investigate whether those procedures cause different inflammatory responses. *Methods*. Twenty patients undergoing craniotomy under general anesthesia and 20 patients undergoing awake function-controlled craniotomy were included in this prospective, observational, two-armed study. Circulating levels of IL-6, IL-8, and IL-10 were determined pre-, peri-, and postoperatively in both patient groups. VAS scores for pain, anxiety, and stress were taken at four moments pre- and postoperatively to evaluate physical pain and mental duress. *Results*. Plasma IL-6 level significantly increased with time similarly in both groups. No significant plasma IL-8 and IL-10 change was observed in both experimental groups. The VAS pain score was significantly lower in the awake group compared to the anesthesia group at 12 hours postoperative. Postoperative anxiety and stress declined similarly in both groups. *Conclusion*. This study suggests that awake function-controlled craniotomy does not cause a significantly different inflammatory response than craniotomy performed under general anesthesia. It is also likely that function-controlled craniotomy does not cause a greater emotional challenge than tumor resection under general anesthesia.

## 1. Introduction

General anesthesia using endotracheal intubation is the standard procedure during brain tumor resection. Vital parameters are monitored and intubation provides a safe airway; drugs ensure analgesia and suppress vegetative reactions. Immobilization is relatively simple, even for patients in an atypical position. However, the use of general anesthesia precludes intraoperative monitoring of higher brain functions, and lesions made to the central nervous system being detected when reversibility of damage control might still be possible. Therefore, awake function-controlled neurosurgery may be beneficial in that respect. During awake craniotomy, the cerebral cortex of the patient is electrically stimulated. This allows the surgeon to properly identify and spare functionally relevant areas of the brain. Awake craniotomy has been shown to be a well-tolerated procedure with minimal side effects. Nevertheless, it is considered to be more challenging for the patient. By allowing for maximal tumor excision while keeping healthy tissue intact, awake craniotomy has the potential for better patient outcomes [1] In such a procedure, the need to provide sufficient analgesia and sedation without interfering with electrophysiological monitoring is essential [2]. 

Before, during, and after craniotomy all patients are confronted with anxiety, stress, and pain. These factors can all negatively influence the perioperative experience. Patients undergoing craniotomy using general anesthesia, however, have to endure additional physical stress factors like intubation, longer hospital stays, and mechanical ventilation [3].

Patient perspectives regarding awake brain surgery have been investigated and adequate preoperative consultation has been found to be essential for patient confidence. In addition, scalp incisions and fixation of pin-holding sites were regarded as major sources of pain and discomfort. Still, the benefits far outweigh those of general anesthesia because awake craniotomy patients report less pain, anxiety, and fear [4, 5]. Even though there are drawbacks, the majority of patients tolerate awake craniotomy very well. 

No study has attempted to compare the inflammatory impact of awake craniotomy versus general anesthesia procedures. Pathological inflammatory states can have far ranging clinical effects and negatively influence a patient's neurological outcome [6–8]. Recent research has demonstrated that cytokine levels can be correlated to the degree of brain tissue manipulation [9]. Plasma cytokine levels could reflect stress-related biochemical pathways after surgery [10–12]. 

Cytokines orchestrate the complex network of cellular interaction that regulate both cellmediated and humoral immunity, as well as the acute phase response [13]. Cytokines are glycopeptide signaling molecules that act at extremely low concentrations, mediating key immune responses. Several cytokines are released during periods of stress, including interleukin-6 (IL-6), IL-8, and IL-10 [14]. IL-6 is a proinflammatory cytokine secreted by T-cells, macrophages, and other cells. IL-6 is involved in both the immune response to trauma and the acute phase response; its targets being T- and B-cells. IL-8 is a chemokine produced mainly by macrophages and epithelial cells and functions to attract neutrophiles towards inflammation sites. These proinflammatory cytokines play a key role in the physiological response to trauma and surgery, whereas IL-10 is an anti-inflammatory cytokine produced by Th2-cells that cause a reduction in proinflammatory cytokine synthesis [15].

Our aim was to investigate whether awake function-controlled craniotomy causes a significantly different inflammatory response than craniotomy performed under general anesthesia. We thought both procedures would create similar inflammatory profiles despite differing anesthesia techniques used. In order to test our hypothesis, plasma levels of IL-6, IL-8, and IL-10 were measured during the pre-, peri-, and postoperative periods in both patient groups. We also noted corresponding subjective outcome parameters for pain, anxiety, and stress to investigate whether performing an awake procedure causes more physical pain and mental duress.

## 2. Patients and Methods

### 2.1. Study Design and Inclusion Criteria

This was a prospective, single centre, two-armed observational study with 40 patients (20 men and 20 women). The protocol was approved by the Medical Ethics Committee of the Erasmus Medical Center, Rotterdam. All procedures were performed in accordance with the Helsinki declaration. Written informed consent was obtained from all patients.

Plasma cytokine determinations were performed blinded, but randomization was limited. The decision to perform either function-controlled awake craniotomy or craniotomy under general anesthesia was determined by the neurosurgeon who based his decision on the intracerebral location of the tumor. The type or size (WHO classification of brain tumors) had no influence on whether or not awake craniotomy was chosen. By proxy patients were allocated to the general anesthesia group unless the location of the tumor warranted the benefits of an awake procedure. Patients with tumors close to functional relevant areas like the motor cortex or areas related to speech require the awake monitoring made possible by the awake craniotomy procedure. By allocating these patients to the awake craniotomy group maximal tumor resection is made possible with a minimal risk of functional neurological damage. 

Eligible patients were >18 years of age and were undergoing craniotomy for an intracerebral tumor. Patients were excluded if they were (1) ASA-classification IV-V, (2) did not provide written informed consent, (3) had a tumor location other than intracerebral, (4) had surgery beginning later than 11:00 a.m., (5) had a disease of the endocrine system or (6) were taking drugs that alter endocrine metabolism (like thyroxine). Noncooperative or noncompliant patients could be withdrawn from the study, as could patients who developed serious adverse effects. 

### 2.2. Anesthesia Procedure

Patients in both groups received 1.5 mg lorazepam on the evening before the surgery. All patients were on a regimen of dexamethasone 4 × 4 mg/day with the first dose given at least one day prior to surgery; regular personal drug regimens were continued during the study. In the awake function-controlled group, 7.5 mg piritramide and 25 mg promethazine were given 30 minutes prior to induction. In the general anesthesia group, premedication consisted of 50 mg promethazine. In both groups propofol was administered for sedation and remifentanil for analgesia. The general anesthesia group received an additional 0.25 mg fentanyl before intubation and placement of the Mayfield clamp. Cis-atracurium was used for muscle relaxation prior to intubation. In order to provide adequate pain control during awake craniotomy patients were infiltrated with bupivacaine 0.375% with adrenaline 1 : 200000 at the site of scalp incision. Postoperatively all patients were offered 4 times one gram paracetamol per day, and if required, supplemental morphine.

### 2.3. Outcome Measures

Patient characteristics, medications used during and after surgery, fluid balance, and duration of surgery were documented. Pain, anxiety, and stress were measured at 12 and 24 hours pre- and postoperatively, using visual analogue scale (VAS) scores (0 = none, 10 = extreme).

EDTA blood samples (7 mL) for cytokine level determinations were collected preoperatively, during the opening and closing of the dura, and 12 and 24 hours postoperatively. Plasma was isolated by centrifugation at 2650 g_max_ for 10 minutes at 20°C; samples were stored at −80°C until assay. 

**Figure 1:**
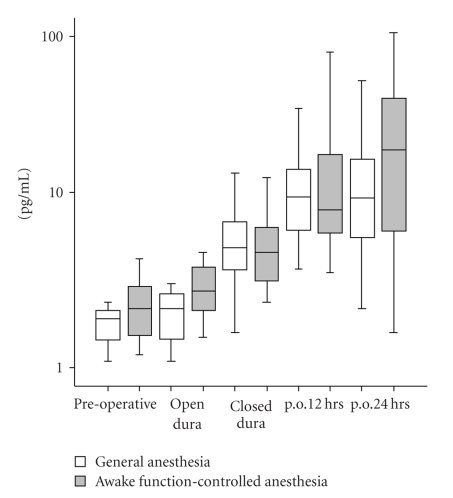
*Box plots of plasma IL-6 levels*. A significant IL-6 level increase is found in both experimental groups F(1.336, 49.416) = 24.148, *P* < .001. No significant plasma IL-6 level difference is found between groups.

**Figure 2:**
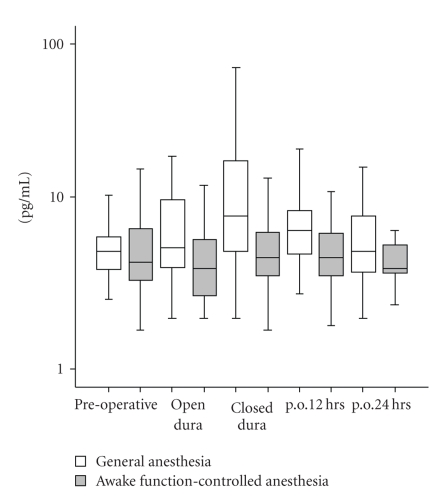
*Box plots of plasma IL-8 levels*. IL-8 levels do not significantly change throughout time for both experimental groups. No significant difference in plasma IL-8 levels is found between groups.

**Figure 3:**
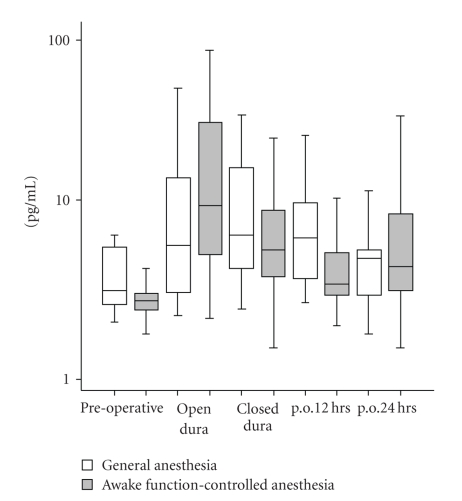
*Box plots of plasma IL-10 levels*. IL-10 levels do not significantly change throughout time for both experimental groups. No significant difference in plasma IL-10 levels is found between groups.

Enzyme immunoassays for the quantitative determination of human IL-6, IL-8, and IL-10 were performed with a sandwich ELISA (Pelikine Compact and additional Pelikine Toolset, Sanquin, Amsterdam, The Netherlands) as described previously [16]. Data were calculated as pg/mL plasma and presented in Figures 1, 2, and 3 as (log) pg/mL.

### 2.4. Statistical Analysis

Data were analyzed using SPSS for Windows, version 16.0.1. The independent sample *t*-test was used to compare means for patient demographics (excluding ASA classification) and perioperative characteristics. The Pearson Chi-square test was used to evaluate differences in ASA classification. All data were reported as the mean (SD), counts, or median (25%–75%).

Sample size was calculated using the O'brien-Shieh Algorithm for the MANOVA repeated measures test. Assuming a medium effect, an effect size of 0.6 was used and a power of 0.8. There were two experimental groups and 5 repetitions. The required a priori sample size computed by this method was 39. 

For the VAS scores and cytokine data the MANOVA test was used. Differences in VAS score or cytokine values between the experimental groups across all time points and interaction between experimental groups and time were analyzed using multivariate repeated measures. Experimental group and time were the independent variables. When Mauchly's Test of Sphericity was significant, the Greenhouse-Geisser test of within subjects effects was used. When a significant difference was found between experimental groups a one-way ANOVA test with posthoc multiple comparisons (Bonferroni correction) was used to analyze the relationship between the cytokines or VAS scores from the first preoperative measurement until 24 hours postoperative. The same Bonferroni correction was employed to analyze differences between experimental groups and time.

A *P*-value < .05 was considered statistically significant.

## 3. Results

Forty patients were included in the study. The awake function-controlled and general anesthesia groups contained 20 patients each, stratified for gender (10 males and 10 females). No significant intergroup differences were observed for age, height, weight, ASA classification, or Hb concentration (Table 1).

Perioperative characteristics are described in Table 2. As expected, the total amount of propofol administered throughout the operation was significantly less in the awake group than in the general anesthesia group. The general anesthesia group also received more crystalloids during the operation. The total amount of remifentanil used in the general anesthesia group was 8.4 ± 5.4 mg. No more than 200 *μ*g of remifentanil was given to the awake craniotomy group.

Plasma concentrations of IL-6, IL-8, and IL-10 during all time points are displayed in Figures 1 through 3. IL-6 level significantly increased with time in both experimental groups (main effect of time: F(1, 49) = 24.1, *P* < .001, observed power = 1.00). However, there were no differences between groups (group-time interaction: F(1, 37) = 1.3, *P* = .3, observed power = 0.20). Furthermore, IL-8 levels did not significantly change with time in both experimental groups (main effect of time: F(1, 48) = 2.2, *P* = .1, observed power = 0.35) and no significant IL-8 differences between groups (group-time interaction: F(1, 37) = 2.8, *P* = .1, observed power = 0.37). The same applied for IL-10 levels, there were no significant change with time in both experimental groups (main effect of time: F(1, 39) = 2.6, *P* = .1, observed power = 0.36) and no significant differences between groups (group-time interaction: F(1, 37) = 0.6, *P* = .4, observed power = 0.12).

There was no significant differences between groups in the amount of postoperative morphine and paracetamol used. The mean subcutaneous postoperative morphine administered in the general anesthesia group was 1.60 (±4.72 mg), while the mean given to the awake group was 2.00 (±5.48 mg). The mean postoperative paracetamol administered to the general anesthesia group was 2100 (±1483 mg), while the mean given to the awake group was 1900 (±1477 mg).

**Figure 4:**
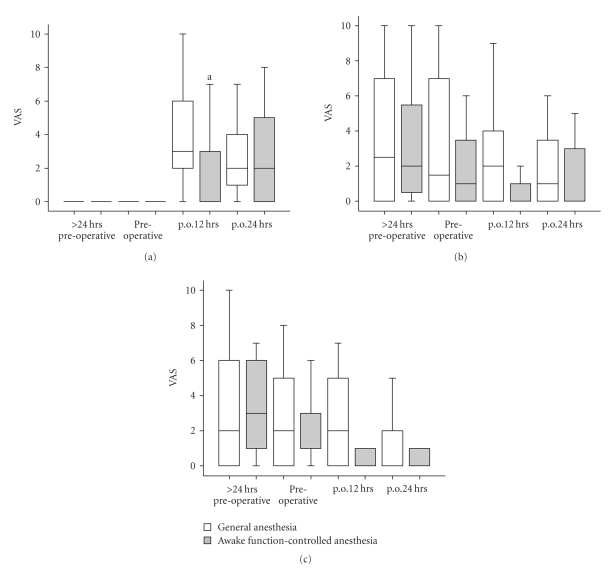
*Box plots of pain, anxiety, and stress*. (a) *Pain*. A significant increase in pain is experienced in both experimental groups F(2.290, 80.165) = 24.642, *P* < .001. A significant difference F(1, 35) = 7.632, *P* = .009 was observed in the awake group compared to the general anesthesia group at “p.o.12 hours” (^a^). (b) *Anxiety*. A significant decrease in anxiety is experienced in both experimental groups F(1.982, 69.362) = 4.637, P = 0.013. (c) *Stress*. A significant decrease in stress is experienced in both experimental groups F(2.426, 84.911) = 7.920, *P* < .001.

Pain increased significantly with time in both experimental groups (main effect of time: F(2, 80) = 24.6, *P* < .001). However, a significant difference between groups (F(1, 35) = 7.6, *P* = .009) was noted with the awake group having less pain at the 12 hours postoperative time point (Figure 4). 

Anxiety significantly decreased with time in both experimental groups (main effect of time: F(2, 69) = 4.6, *P* = .013) and there was a significant stress decrease with time in both experimental groups (main effect of time: F(2, 85) = 7.9, *P* < .001).

## 4. Discussion

We believe we are the first to compare the cytokine profiles of awake and general anesthesia craniotomy groups. Cytokine release is also a known physical reaction to tissue damage. The influence of surgery on cytokine plasma levels has been addressed during several studies. There is a great amount of evidence linking IL-6 to the degree of surgical trauma [17–22]. In addition, there are also studies that establish a clear relationship between dynamic IL-6 changes and cortisol plasma levels during the perioperative period [11, 23]. The nonsignificant differences in IL-6 levels between groups found during this experiment suggest from an immunological perspective that both procedures are likely to be similarly stressful for the body. However, the low and medium observed power of our negative findings requires a larger patient group to provide more certainty. 

It is interesting to note the significant plasma IL-6 increase despite the exact dexamethasone 4 × 4 mg/day regime given to both experimental groups. Another study investigating the effects of dexamethasone produced different results. Morariu et al. found that after receiving dexamethasone (1 mg/kg) before anesthesia induction, plasma levels of both IL-6 and IL-8 were significantly reduced, while levels of IL-10 increased perioperatively [24]. 

Our finding that there was a significant plasma IL-6 increase throughout time for both experimental groups and a significant increase in reported pain can be partially explained by the expected increase in pain after tissue damage. It is noteworthy that an increasing pain trend matches the increasing IL-6 tendency observed. The important role interleukin-6 plays in nociception and the pathophysiology of pain during a variety of different conditions might explain this trend [25]. A study done with rat models observed that higher IL-6 concentrations were linked to more intense hyperalgesia [26]. 

Recently, plasma IL-8 has been measured as a key mediator for neuroinflammation in patients with severe traumatic brain injuries [27]. Central venous plasma IL-8 levels were significantly lower in survivors than in nonsurvivors. In our study, the insignificant in-between and within-subject plasma IL-8 change in both experimental groups was unexpected. Due to IL-8's presence in neutrophils, microglia, astrocytes and endothelial cells of the brain [28–31] we expected damaged brain tissue to cause an increased release of IL-8 over time from these sources. However, the studies involving traumatic brain injury patients contain a different patient population then ours and different confounders. The additional hypoxia and ischemia experienced in these severely injured traumatic brain injury patients can be attributed to shock and resulting hypoperfusion and might account for increased plasma IL-8 levels [32]. 

Awake craniotomy is considered a stressful procedure. It seems logical that being awake while a neurosurgeon removes pathological brain tissue would lead to a more intense emotional response than undergoing the same procedure under general anesthesia. However, perhaps good psychological support and active coping mechanisms may actually make awake craniotomy less stressful for the patient. This might be due to the awake group having decreased feelings of dependency and loss of control than those in the general anesthesia group.

Our results show that patients undergoing awake function-controlled craniotomy experience less 12 hours postoperative pain than their general anesthesia counterparts. The intensive preoperative consultation patients received might have influenced results due to the subjective nature of the VAS scoring system [33]. It could be argued that perioperative medication may also have influenced VAS score results. Patients who underwent awake function controlled craniotomy received 25 mg of promethazine and 7.5 mg of piritramide 30 minutes before surgery. In comparison, general anesthesia patients received 50 mg of promethazine and two boluses of fentanyl, one prior to induction and another prior to placement of the Mayfield clamp. Piritramide and fentanyl are both opiates with additive sedative and euphoric properties. They are also accepted drugs for surgical procedures like craniotomy [34]. Additionally, the seven and six hours half life of piritramide and fentanyl make them unlikely to affect the first postoperative VAS score measurement taken at 12 hours postoperative [35, 36]. We think that the local anesthesia provided by bupivacaine infiltration at the site of scalp incision was the primary reason why VAS scores were significantly lower in the awake group. 

The differing nature of awake craniotomy and general anesthesia techniques requires a larger amount opiates to be given to the general anesthesia group. There is some evidence that opiates can modulate the immune system [37–39]. However, our results reveal similar pro- and anti-inflammatory profiles for both groups with no significant difference having been found between groups. It is still important to consider that the larger opiate amount given to the general anesthesia group could have altered its immunological profile. However, the aim of this study is to compare the inflammatory profile of two different anesthesia techniques. General anesthesia cannot be performed without a greater amount of opiates being used by the anesthesiologist. 

The smaller amount of propofol administered to the awake group is due to the reduced need for sedation during the awake craniotomy procedure. On the other hand, the larger amount of crystalloids given to the general anesthesia group can be explained by the need to counteract the vasodepressive properties of propofol (Table 2).

A limitation of our study is that for ethical reasons allocation of patients to one group or another could not be randomized. This restriction could bias our results. However keeping the previously mentioned limitations in mind, the plasma levels of pro- and anti-inflammatory cytokines measured during this study suggests that awake function-controlled craniotomy does not cause a significantly different inflammatory response than craniotomy performed under general anesthesia. Furthermore, the nonsignificant difference in subjective outcome parameters for pain (with exception 12 hours postoperative), anxiety, and stress insinuates that both procedures are equally mentally challenging. Therefore, it is likely that function-controlled craniotomy does not cause a greater inflammatory insult or emotional challenge than patients undergoing tumor resection using general anesthesia.

## Figures and Tables

**Table 1:** Patient demographics.

	General anesthesia	Function-controlled
Age (years)	48 ± 15.4	44 ± 13.2
Gender m/f	10/10	10/10
Height (cm)	174 ± 11.3	176 ± 9.6
Weight (kg)	74 ± 16.5	81 ± 14.7
ASA classification 1/2/3 (number of patients)	9/10/1	5/15/0
Hb concentration (mmol/L)	9.3 ± 1	9.0 ± 0.6

Data presented as mean ± SD

**Table 2:** Perioperative characteristics.

	General anesthesia	Function-controlled
Propofol during operation (mg)	3277 ± 1632	673 ± 313^a^
Operation time (min)	327 ± 104	275 ± 56
Blood loss during operation (L)	400 (300–500)	450 (300–600)
Colloids during operation (L)	500 (500–500)	500 (0–500)
Colloids after operation (L)	0.05 ± 0.2	0.1 ± 0.4
Crystalloids during operation (L)	3.7 ± 2.0	1.6 ± 0.7^a^
Crystalloids after operation (L)	2.0 ± 1.0	2.0 ± 0.9
Urine during operation (mL)	1620 (1043–2050)	1042 (480–1483)^b^
Urine after operation (mL)	1759 ± 836	1668 ± 620
Remifentanil	8.4 ± 5.4 mg	200 *μ*g*
Postoperative paracetamol (mg)	2100 ± 1483	1900 ± 1477
Postoperative morphine (mg)	1.60 ± 4.72	2.00 ± 5.48

Function-controlled versus general anesthesia significantly decreased: ^a^ *P* < .001 and ^b^ *P* = .004. Data presented as mean ± SD and median (25%–75%).

*Maximum total amount of boluses given.
